# Trainable joint bilateral filters for enhanced prediction stability in low-dose CT

**DOI:** 10.1038/s41598-022-22530-4

**Published:** 2022-10-20

**Authors:** Fabian Wagner, Mareike Thies, Felix Denzinger, Mingxuan Gu, Mayank Patwari, Stefan Ploner, Noah Maul, Laura Pfaff, Yixing Huang, Andreas Maier

**Affiliations:** Pattern Recognition Lab, Friedrich-Alexander-Universität Erlangen-Nürnberg, 91058 Erlangen, Germany

**Keywords:** Image processing, Machine learning

## Abstract

Low-dose computed tomography (CT) denoising algorithms aim to enable reduced patient dose in routine CT acquisitions while maintaining high image quality. Recently, deep learning (DL)-based methods were introduced, outperforming conventional denoising algorithms on this task due to their high model capacity. However, for the transition of DL-based denoising to clinical practice, these data-driven approaches must generalize robustly beyond the seen training data. We, therefore, propose a hybrid denoising approach consisting of a set of trainable joint bilateral filters (JBFs) combined with a convolutional DL-based denoising network to predict the guidance image. Our proposed denoising pipeline combines the high model capacity enabled by DL-based feature extraction with the reliability of the conventional JBF. The pipeline’s ability to generalize is demonstrated by training on abdomen CT scans without metal implants and testing on abdomen scans with metal implants as well as on head CT data. When embedding RED-CNN/QAE, two well-established DL-based denoisers in our pipeline, the denoising performance is improved by 10%/82% (RMSE) and 3%/81% (PSNR) in regions containing metal and by 6%/78% (RMSE) and 2%/4% (PSNR) on head CT data, compared to the respective vanilla model. Concluding, the proposed trainable JBFs limit the error bound of deep neural networks to facilitate the applicability of DL-based denoisers in low-dose CT pipelines.

## Introduction

Minimizing patient dose in computed tomography (CT) is necessary to avoid radiation-related diseases^1^, especially with the number of conducted diagnostic CT scans increasing every year^2^. Low-dose CT acquisitions reduce patient dose^3,4^ but contain higher noise levels in the measured data^5,6^. To enhance the image quality of low-dose CT acquisitions, image-based denoising approaches have been proposed, which aim to preserve clinically relevant features compromised with noise. Classical approaches are based on physically motivated conventional filters, considering the inherent properties of the image features^7–11^. Although such filters produce reliable results through a clear algorithmic formulation, their performance is restricted by a limited capability to extract complex features. In addition, conventional filters often require hyperparameters that have to be tuned by hand. Therefore, deep learning (DL)-based denoising methods gained interest due to their flexibility, strong performance, and data-driven optimization^12–17^. However, deep neural networks usually do not robustly generalize beyond their finite training data distribution, which so far limits clinical applications of DL-based denoising for low-dose CT^18,19^.

Previously, Maier et al. proved that including physical knowledge in terms of known operators in neural networks reduces the absolute error bound of the model^20–22^. Consequently, different image processing pipelines were proposed, employing physical assumptions about noise characteristics to leverage prediction reliability of DL-based methods in the context of image denoising^23,24^. The joint bilateral filter (JBF) is a conventional denoising filter that allows edge-preserving denoising while considering additional information in terms of a guidance image during its filter operation. Imitating the JBF with a shallow convolutional network led to a reduction of trainable parameters in the JBFnet^23^ and the MJBF architecture^25^. Although both network architectures are inspired by the JBF operation, they both learn filter operations through fully convolutional neural networks with a relatively large number of free parameters compared to the JBF. Therefore, both architectures can learn any possible filter kernels and are not enforced to perform the well-know JBF operation, which raises questions on data integrity and interpretability likewise to other DL methods^24^. A different approach employs a custom bilateral filter approximation built from neural network building blocks that can be optimized^26^, but it does not allow integration of additional learned information into the filter process. Other works presented methods to find optimal filter^27^ or training^28^ hyperparameters by predicting them through external neural networks. However, such approaches do not allow for direct integration into DL models as they can not compute gradients toward those hyperparameters.

In our previous work, we presented a trainable bilateral filter with competitive denoising performance that can be included in a differentiable pipeline and optimized in a data-driven fashion^29^. However, the prediction of bilateral filter layers is solely dependent on three learned spatial parameters and one intensity parameter^9^. Therefore, the bilateral filter operation is conceptually different from the joint bilateral filter algorithm, as JBFs allow considering additional information in terms of a guidance image in their denoising algorithm^30^. In this work, we extend our research on bilateral filtering by proposing a fully differentiable, trainable joint bilateral filter that allows denoising using a learned guidance image which broadens its applicability. Our filter layer derives analytical gradients toward the filter input, the image guide, and all filter parameters to achieve differentiability and enable data-driven optimization. Guidance images are estimated using two well-established denoising algorithms: RED-CNN^12^, an encoder–decoder architecture achieving competitive performance in recent works^31,32^, and Quadratic Autoencoder (QAE)^13^, employing quadratic neurons. Our proposed hybrid filter model bridges the gap between deep neural networks’ high model capacities and the robustness of conventional denoising filters due to the well-defined, restricted influence of the learned guide.

### Contributions

Our contributions are threefold. First, we propose a GPU-based, trainable JBF based on an analytical gradient that can be included in any differentiable pipeline. To the best of our knowledge a directly trainable JBF was never presented before. Second, we introduce a hybrid denoising pipeline combining the flexibility of deep neural networks with the robustness of the trainable JBF. Third, we demonstrate the robustness of our model on abdomen CT scans containing metal, with metal not being present in the training data distribution and on out-of-domain head CT scans. Our hybrid JBF-based denoising setting improves the prediction reliability of existing DL-based models with limited computational overhead.

## Methods

Artificial neural networks are generally trained via gradient descent optimization by minimizing a loss metric *L* calculated from network predictions to fulfill a desired task^33^. This requires calculating the derivative of the loss *L* with respect to each trainable model parameter to iteratively update the network during training.

**Figure 1:**
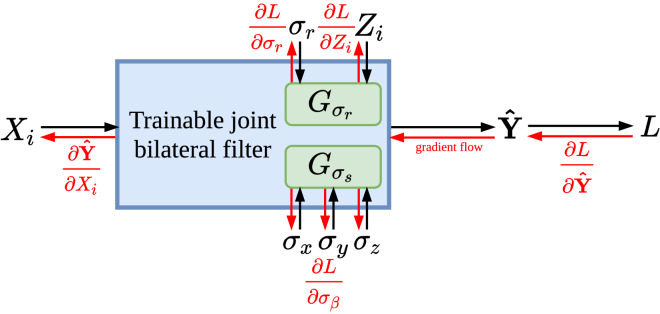
Illustration of the proposed trainable joint bilateral filter layer. In the forward pass (black arrows), the input $X_i$ is filtered using parameters $\sigma _\gamma$ $(\gamma \in \{x, y, z, r\})$ and the guidance image $Z_i$ to predict the denoised image ${\varvec{\hat{Y}}}$. The model’s loss is indicated as *L*. Analytical derivatives are calculated in the backward pass (red arrows) toward filter input, guide, and parameters.

In this section, the analytical gradient of the proposed trainable JBF layer with respect to filter input, guidance image, and filter parameters is derived as the algorithmic contribution of our work. Figure 1 illustrates the general working principle of the denoising layer. In the forward filter operation an input image is convolved with two Gaussian kernels, namely one spatial and one range kernel. The spatial kernel averages pixels within the distance of the filter kernel like a conventional Gaussian filter that smooths the image. An additional, so-called range kernel weighs the influence of pixels from the neighborhood dependent on their intensity difference to the filtered pixel to prevent blurring of edges. The JBF derives its range kernel on an external guidance image which allows employing additional information during the filter operation.

In the following, bold letters are used to indicate vectors. According to Petschnigg et al.^30^ the JBF operation is defined as

1 $$\begin{aligned} {\hat{Y}}_k = \frac{1}{w_k} \underbrace{\sum\nolimits_{n \in {\mathcal {N}}} G_{\sigma _s}({\mathbf {p}}_k - {\mathbf {p}}_n) G_{\sigma _r}(Z_k - Z_n) X_n}_{\begin{array}{c} =:\,\alpha _k \end{array}} \end{aligned}$$

and the normalizing factor $w_k$ as

2 $$\begin{aligned} w_k := \sum _{n \in {\mathcal {N}}} G_{\sigma _s}({\mathbf {p}}_k - {\mathbf {p}}_n) G_{\sigma _r}(Z_k - Z_n), \end{aligned}$$

with the denoised prediction $\varvec{{\hat{Y}}}$ indexed by $k \in {\mathbb {N}}$, the noisy input image ${\mathbf {X}}$ in the voxel neighborhood $n \in {\mathcal {N}}$ around *k*, and a guidance image ${\mathbf {Z}}$. Guidance images should provide additional information to the filter operation and can be, e.g., additional images paired with the filter input or learned predictions from a neural network as later introduced in this work. The Gaussian intensity range kernel

3 $$\begin{aligned} G_{\sigma _r}(c) := \exp \left( -\frac{c^2}{2\sigma _r^2}\right) \end{aligned}$$

is derived from intensity differences on the guidance image ${\mathbf {Z}}$ and enforces edge sensitivity of the filtering operation. A second, spatial filter kernel $G_{\sigma _s}$ weights voxels according to their spatial distance derived from the positions ${\mathbf {p}}_k \in {\mathbb {N}}^d$ and ${\mathbf {p}}_n \in {\mathbb {N}}^d$ with $d=3$ for three-dimensional filtering

4 $$\begin{aligned} G_{\sigma _s}({\mathbf {c}}) = \prod _{s \in \{x,y,z\}} \exp \left( -\frac{c_s^2}{2\sigma _s^2}\right) . \end{aligned}$$

DL pipelines require gradient calculation of the loss function *L* with respect to each trainable parameter to enable data-driven optimization. We can calculate the gradient for our joint bilateral filter layer by using the chain rule

5 $$\begin{aligned} \frac{\partial L}{\partial \sigma _\gamma } = \frac{\partial L}{\partial \varvec{{\hat{Y}}}} \frac{\partial \varvec{{\hat{Y}}}}{\partial \sigma _\gamma } = \sum _k \frac{\partial L}{\partial {\hat{Y}}_k} \frac{\partial {\hat{Y}}_k}{\partial \sigma _\gamma } \end{aligned}$$

with the four kernel widths $\sigma _\gamma$ representing the only trainable weights of the proposed layer when filtering in three dimensions $(\gamma \in \{x, y, z, r\})$. The derivative of the loss function with respect to the filter prediction $\frac{\partial L}{\partial \varvec{{\hat{Y}}}}$ is provided by the backpropagation of the loss through differentiable operations applied on the JBF layer output, e.g., subsequent convolutional layers or the loss function itself. The term $\frac{\partial {\hat{Y}}_k}{\partial \sigma _\gamma }$ can be written using the definition of the joint bilateral filter algorithm from Eq. (1) together with the product and chain rule of differentiation

6 $$\begin{aligned} \frac{\partial {\hat{Y}}_k}{\partial \sigma _\gamma } = - w_k^{-2} \alpha _k \frac{\partial w_k}{\partial \sigma _\gamma } + w_k^{-1} \frac{\partial \alpha _k}{\partial \sigma _\gamma }, \end{aligned}$$

the partial derivatives

7 $$\begin{aligned} \frac{\partial w_k}{\partial \sigma _\gamma }&= \sum _{n \in {\mathcal {N}}} \frac{\partial }{\partial \sigma _\gamma } G_{\sigma _s}({\mathbf {p}}_k - {\mathbf {p}}_n) G_{\sigma _r}(Z_k - Z_n), \end{aligned}$$

8 $$\begin{aligned} \frac{\partial \alpha _k}{\partial \sigma _\gamma }&= \sum _{n \in {\mathcal {N}}} X_n \frac{\partial }{\partial \sigma _\gamma } G_{\sigma _s}({\mathbf {p}}_k - {\mathbf {p}}_n) G_{\sigma _r}(Z_k - Z_n), \end{aligned}$$

and the Gaussian terms

9 $$\begin{aligned} \frac{\partial }{\partial \sigma _\gamma } G_{\sigma }(c) = G_{\sigma }(c) \frac{c^2}{\sigma _\gamma ^3}. \end{aligned}$$

In addition, the derivative of the loss with respect to each input voxel $X_i$ of the joint bilateral filter yields

10 $$\begin{aligned} \begin{aligned} \frac{\partial L}{\partial X_i} &=\frac{\partial L}{\partial \varvec{{\hat{Y}}}} \frac{\partial \varvec{{\hat{Y}}}}{\partial X_i} = \sum _k \frac{\partial L}{\partial {\hat{Y}}_k} \frac{\partial {\hat{Y}}_k}{\partial X_i}\\ &=\sum _k \frac{\partial L}{\partial {\hat{Y}}_k} w_k^{-1} G_{\sigma _s}({\mathbf {p}}_k - {\mathbf {p}}_i) G_{\sigma _r}(Z_k - Z_i) \end{aligned} \end{aligned}$$

using the definition of the JBF from Eq. (1). This gradient calculation to the filter input is required to allow including the filter as a trainable layer into a differentiable pipeline. The derivative of *L* with respect to each voxel of the guidance image $Z_i$ can be calculated as

11 $$\begin{aligned} \begin{aligned} \frac{\partial L}{\partial Z_i} =&\sum _k \frac{\partial L}{\partial {\hat{Y}}_k} \frac{\partial {\hat{Y}}_k}{\partial Z_i} = \sum _k \frac{\partial L}{\partial {\hat{Y}}_k} \left( - w_k^{-2} \alpha _k \frac{\partial w_k}{\partial Z_i} + w_k^{-1} \frac{\partial \alpha _k}{\partial Z_i} \right) \end{aligned} \end{aligned}$$

where the following two cases must be distinguished: Case 1 derives gradients to arbitrary voxels located in the filter neighborhood ($k \ne i$) of the guidance image. In contrast, Case 2 defines the gradient to the center voxel ($k = i$) of the respective filter window.

### Case 1: ($\varvec{k \ne i}$)

12 $$\begin{aligned} \left. \frac{\partial w_k}{\partial Z_i}\right| _{k \ne i}&= \,G_{\sigma _s}({\mathbf {p}}_k - {\mathbf {p}}_i) G_{\sigma _r}(Z_k - Z_i) \frac{Z_k - Z_i}{\sigma _r^2}\\ \left. \frac{\partial \alpha _k}{\partial Z_i}\right| _{k \ne i}&= \,G_{\sigma _s}({\mathbf {p}}_k - {\mathbf {p}}_i) G_{\sigma _r}(Z_k - Z_i) \frac{Z_k - Z_i}{\sigma _r^2} X_i \end{aligned}$$

### Case 2: ($\varvec{k = i}$)

13 $$\begin{aligned} \left. \frac{\partial w_k}{\partial Z_i}\right| _{k = i}&= \,\sum _{n \in {\mathcal {N}}} G_{\sigma _s}({\mathbf {p}}_i - {\mathbf {p}}_n) G_{\sigma _r}(Z_i - Z_n) \frac{Z_n - Z_i}{\sigma _r^2}\\ \left. \frac{\partial \alpha _k}{\partial Z_i}\right| _{k = i}&= \,\sum _{n \in {\mathcal {N}}} G_{\sigma _s}({\mathbf {p}}_i - {\mathbf {p}}_n) G_{\sigma _r}(Z_i - Z_n) \frac{Z_n - Z_i}{\sigma _r^2} X_n. \end{aligned}$$

We calculate the analytical gradients in the backward pass of a fully trainable JBF using the CUDA binding of the *PyTorch* deep learning framework^34^ to leverage computational performance. The processing time of one $512 \times 512$ image using $5 \times 5$/$11 \times 11$ pixel kernel windows is around $1.8\,\text{ms}$/$8.0\,\text{ms}$ on the GPU and $69\,\text{ms}$/$350\,\text{ms}$ on the CPU. In comparison, *torch.nn.Conv2d* layers (*PyTorch*) approximately require $0.1\,\text{ms}$/$0.2\,\text{ms}$ (GPU) and $8\,\text{ms}$/$20\,\text{ms}$ (CPU) for processing the single channel image. For both layers, gradient calculations have comparable run times as their forward passes. All run times were estimated by averaging 50 repeated forward/backward passes through the respective layers using a *NVIDIA Quadro RTX 4000* GPU. Please note that run times can strongly vary depending on the used hardware.

The filter window size of the JBF is chosen dynamically dependent on the spatial kernel sizes as $5 \cdot \sigma _s$. This ensures that $>98\,\%$ of the Gaussian filter kernel mass is contained by the filter window which turned out to be a reasonable trade-off between accuracy and computational complexity of the algorithm.

Our filter layer is publicly available at https://github.com/faebstn96/trainable-joint-bilateral-filter-source and can be installed via the well-known Python Package Index (PyPI) as plug-and-play PyTorch layer. In addition, our code repository contains example scripts and a test script that compares the implementation of the analytical gradients with numerical gradient approximations using the *torch.autograd.gradcheck* function to make sure the filter derivative is correctly implemented.

## Experimental setup

### Denoising pipeline

**Figure 2:**
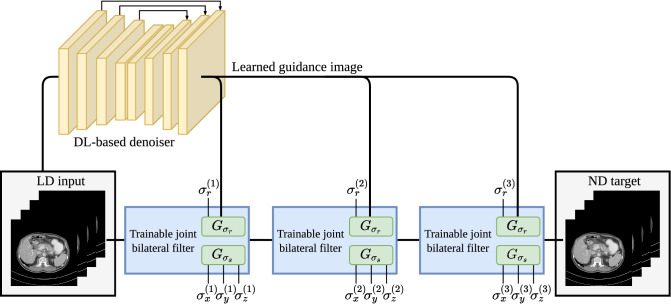
The investigated JBF-based denoising pipeline consists of three stacked trainable JBF layers to iteratively remove noise from the low-dose input reconstruction. Pre-trained RED-CNN and QAE models are employed as DL-based denoiser to predict guidance images. The model is trained supervised on the training domain and tested on CT data from other domains to investigate robustness properties of the pipeline. Indices $(\nu )$ with $\nu \in \lbrace 1, 2, 3\rbrace$ name the individual trainable JBF layers.

Our denoising pipeline, illustrated in Fig. 2, is built on three consecutive trainable JBF layers. The iterative composition of filtering blocks is inspired by the design of the deep convolutional architecture JBFnet^23^ and our previous experimental findings on using multiple stacked bilateral filters^29^ which improved performance compared to employing only a single denoising step. The three trainable JBFs add in total twelve independently trainable parameters to the denoising model. The forward pass of each filter layer is calculated as written in Eq. (1). A guidance image is predicted from a deep convolutional network and used to derive the weighting of the intensity range kernel $G_{\sigma _r}$ in each JBF. Multiple network configurations are presented in the following, investigating the influence of JBF layers on the denoised prediction.

### Experiments

Our experiments are particularly designed to investigate the prediction robustness of hybrid JBF + DL-based denoising models compared to the respective vanilla DL model. We perform experiments with two different well-established low-dose CT denoising architectures predicting the guidance image: RED-CNN^12^ and QAE^13^. In all our experiments, we train the two reference models independently as described in their works until full convergence of the validation loss, occurring after up to 300 epochs. Subsequently, we place the models in our denoising pipeline and optimize the JBFs for additional 200 epochs until convergence of the validation loss. Both trained vanilla deep neural networks are used as performance reference. We use the mean squared error loss and two separate Adam optimizers for $\sigma _r$ ($l_r = 1 \cdot 10^{-2}$) and $\sigma _s$ ($l_r = 5 \cdot 10^{-4}$) during training as both sets of parameters define filter kernels that act on independent scales. However, additional experiment where we used only a single Adam optimizer converged to very similar sets of filter parameters within comparable numbers of optimization steps. Therefore, we conclude that the network convergence is not overly sensitive to learning rate configurations when using an Adam optimizer.

### Data

**Figure 3:**
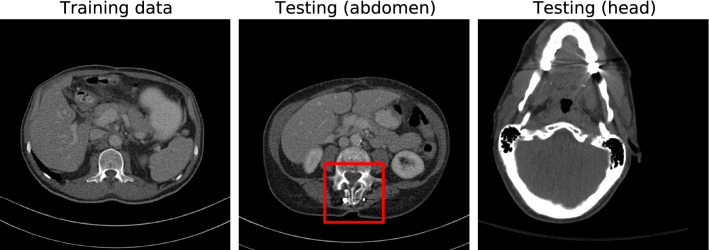
Exemplary slices from the training (left, abdomen without metal) and two testing (middle, right) data sets. The abdomen test patients contain regions with metal implants like the one highlighted in red. The reconstruction window is $[-150, 500]\,\text{HU}$.

All used abdomen and head CT scans are from the public *TCIA Low Dose CT Image and Projection* data set (Version 4)^35^, containing paired low-dose (25% dose) and high-dose CT volumes. The goal of our experiment is to quantitatively evaluate the robustness of the introduced denoising models and compare them with the vanilla DL-based denoising models RED-CNN and QAE. Therefore, we manually split the abdomen data into two domains. First, patients without metal pieces and second reconstructions containing pieces of metal like implants or catheters that appear as bright regions due to their strong x-ray absorption. Only data from the first domain not containing metal is used for training (21 scans) and validation (two scans). Subsequently, we test our models on the previously unseen metal domain scans (24 scans) to evaluate how the different architectures can handle examples that are insufficiently represented by the training data domain. As the metal pieces are usually located in small sub-volumes of the reconstructions, we additionally define 17 three-dimensional regions of interest (ROIs) that are evaluated separately to get more expressive results on the sensitivity to the out-of-domain features. The coordinates of all 17 ROIs are provided in the supplementary material together with exemplary abdomen slices containing the respective ROIs to facilitate reproducibility. Additionally, we test our models on data from a separate domain, namely head CT scans (20 scans), to investigate prediction robustness on a different anatomy. Figure 3 shows example slices from the training and testing data sets with a highlighted abdomen ROI containing metal parts. Note that all scans are directly taken from the public data set without further modification such that they well represent clinical routine head and abdomen CT acquisitions of patients with and without metal implants^35^.

## Results

### Quantitative results

We present quantitative denoising results on the entire abdomen test data set and only on the abdomen ROIs containing metal pieces in Table 1. Performance metrics for the investigated out-of-domain head CT data set are listed in Table 2. The three established image quality metrics root-mean-square error (RMSE), peak signal-to-noise ratio (PSNR), and structural similarity (SSIM)^36^ are calculated to compare model prediction with their respective high-dose target reconstruction. RMSE and PSNR particularly assess deviations from the target image intensities, whereas SSIM aims to imitate human perception to compare image content. We found that all performance differences between vanilla and respective JBF-based model in Table 1 are significant based on a Wilcoxon signed-rank test^37^ on a *p*-value $p < 0.005$. The Wilcoxon signed-rank test is particularly suited to test the paired model predictions at hand without presuming an underlying statistical model.

**Table 1:** Quantitative denoising results on the full abdomen scans containing metal implants as well as on 17 ROIs that contain metal parts.

	Full metal data			Metal ROIs		
	RMSE [HU] $\downarrow$	PSNR $\uparrow$	SSIM $\uparrow$	RMSE [HU] $\downarrow$	PSNR $\uparrow$	SSIM $\uparrow$
Low-dose CT	18.6 ± 6.1	41.51 ± 2.13	0.9512 ± 0.031	26.7 ± 15.9	38.98 ± 4.28	0.9594 ± 0.030
RED-CNN^12^	**12.0** ± **3.9**	**45.38** ± **1.93**	**0.9816** ± **0.012**	24.6 ± 13.1	39.68 ± 3.75	0.9788 ± 0.015
RED+JBFs	12.7 ± 4.5	45.01 ± 2.02	0.9797 ± 0.016	**22.1** ± **14.2**	**40.82** ± **4.27**	**0.9797** ± **0.013**
QAE^13^	44.7 ± 36.1	43.17 ± 4.49	0.9811 ± 0.014	837.8 ± 478.3	14.88 ± 10.92	0.6967 ± 0.232
QAE+JBFs	**14.5** ± **5.4**	**44.61** ± **2.23**	**0.9827** ± **0.010**	**150.9** ± **87.5**	**26.99** ± **7.19**	**0.9075** ± **0.062**

The metrics are averaged over all test patients or all test ROIs and provided as $\text{mean} \pm \text{std}$. The respectively better performing network modification is highlighted in bold.

**Table 2:** Quantitative denoising results on the full head CT data set.

	Head CT data		
	RMSE [HU] $\downarrow$	PSNR $\uparrow$	SSIM $\uparrow$
Low-dose CT	5.3 ± 1.1	38.36 ± 1.58	0.9338 ± 0.017
RED-CNN^12^	5.1 ± 0.6	38.42 ± 0.88	0.9646 ± 0.006
RED+JBFs	**4.8** ± **0.6**	**39.01** ± **0.98**	**0.9662** ± **0.006**
QAE^13^	44.8 ± 40.9	37.30 ± 2.58	0.9679 ± 0.007
QAE+JBFs	**9.8** ± **6.4**	**38.97** ± **1.77**	**0.9695** ± **0.007**

The metrics are averaged over all test patients and provided as $\text{mean} \pm \text{std}$. The respectively better performing network modification is highlighted in bold.

Whereas the hybrid JBF layer-based pipelines perform comparably to the vanilla deep denoising models over the entire abdomen test data, an explicit performance improvement is recognized on the 17 abdomen ROIs as well as on the head CT scans on all three investigated image quality metrics. Both JBF-based pipelines decrease the RMSE by 10%/82% and improve the PSNR and SSIM by 3%/81% and 0.1%/30% around the out-of-training-domain metal features compared to the vanilla RED-CNN and QAE respectively. The denoising performance on the head CT data is improved by 6%/78% (RMSE), 2%/4% (PSNR), and 0.1%/0.1% (SSIM).

### Qualitative results

**Figure 4:**
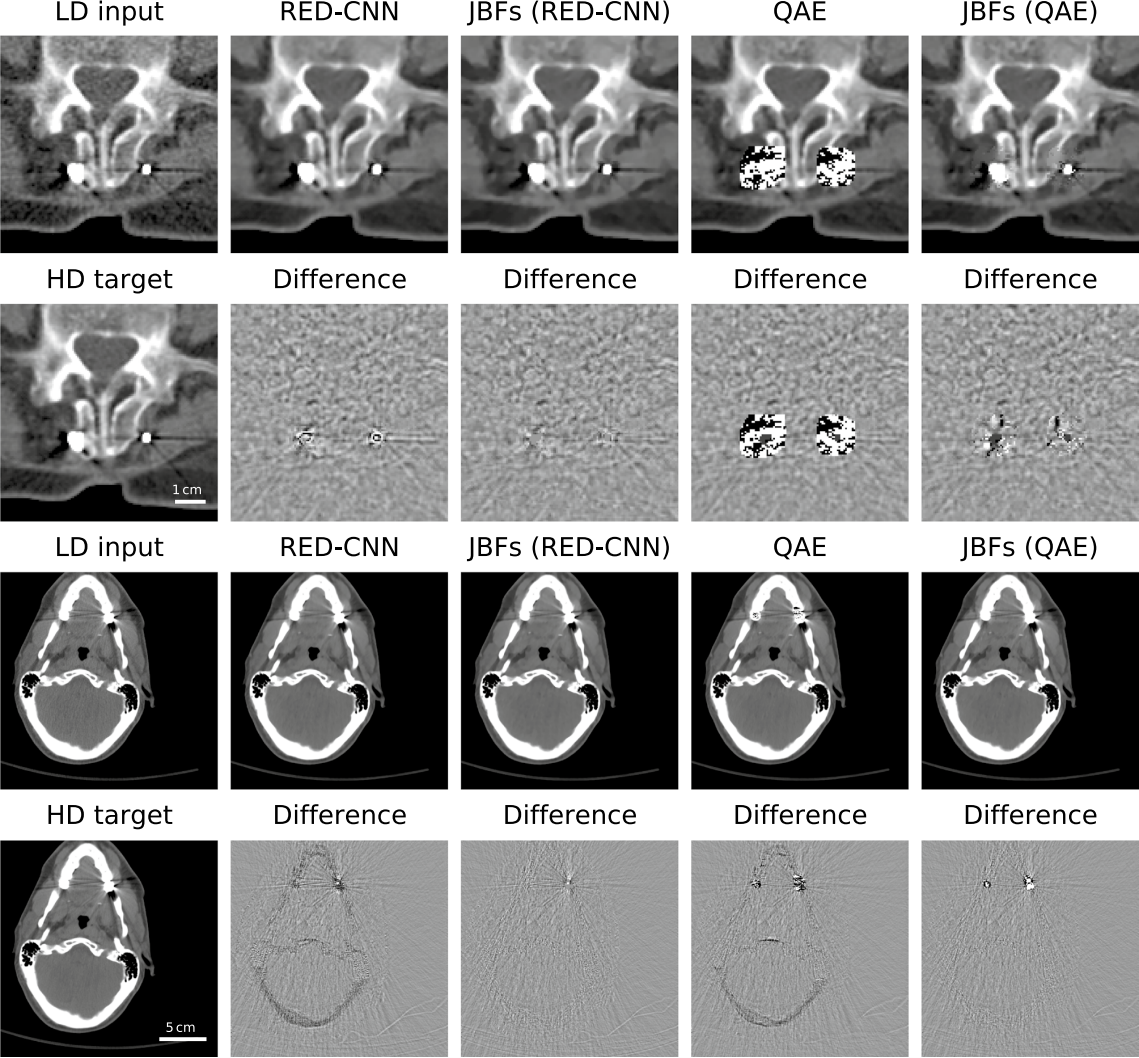
Qualitative denoising results on the ROI highlighted in Fig. 3 and on a head CT slice. Difference images are calculated between model prediction and high-dose (HD) target and are shown in the window $[-50, 50]\,\text{HU}$ for abdomen data and $[-100, 100]\,\text{HU}$ for head data. The reconstruction window is $[-150, 500]\,\text{HU}$. Our hybrid models visually outperform the respective vanilla deep models.

Visual results on one ROI and a head CT slice are displayed in Fig. 4. Provided difference images between model prediction and high-dose target particularly highlight disturbed features and erroneous predictions. Intensity distortions in close proximity to metal implants and in the skull region can be recognized for the RED-CNN, which get almost entirely removed using the RED-CNN prediction as guidance image in a JBF-based setting. Here, in particular the intensity shifts visible as shadows of the skull in the difference images of the vanilla model prediction are fully restored by the proposed hybrid JBF-based model.

The QAE predicts strong artifacts that are visible in the abdomen intensity images and difference images surrounding metal implants. Using such predictions as an image guide in a JBF-based pipeline produces results that visually look much closer to the high-dose target where features like the shape of metal pieces or the adjacent anatomy are visible. Further, intensity distortions in QAE predictions on the head CT data set are removed using the combined QAE+JBFs filtering approach. Only regions around the dental crowns with heavy metal reconstruction artifacts remain disturbed.

## Discussion

Although one could simply add abdomen scans containing metal pieces or head CT data to the training data set to improve denoising performance, our experiment is particularly designed to evaluate and quantify robustness to real CT data that is underrepresented in the training data. Our experiment, therefore, mimics the present clinical scenario where a model is only trained on a limited number of scans but must also handle differing anatomies or scanning parameters. The denoising performance of a JBF depends on an optimal intensity range kernel $G_{\sigma _r}$ to avoid blurring edges. Here, the proposed pipeline can benefit from the guidance image that is predicted by a deep model that is capable of employing global image features to facilitate extracting sharp edges needed for the filter kernel computation. In case of prediction failures like in regions around metal implants or at the skull, the intensity range kernel contribution is either over- or underestimated. This results in over- or under-smoothing of the respective image region but is always based on the local content of the input image. Therefore, the intensity range kernel design of Gaussian shape prevents the output from large prediction errors by design.

In our conducted experiments, pre-trained denoising networks predict the guidance images that are input to the JBF layers. We performed additional experiments, training the JBFs together with the denoising networks in a combined end-to-end setting. Although this setting enhanced performance within the training data domain, we did not recognize explicit performance improvements in terms of robustness on the investigated out-of-training-domain data sets. Eventually, we did not design our experiments to answer the question how a guidance image that is optimized for JBFs is handled in the training data domain but we particularly want to investigate how JBFs handle the displayed artifacts predicted by the denoising networks as the primary goal of our study.

JBF-based pipelines almost entirely prevent the predictions from artifacts introduced by the DL-based models but the combined QAE+JBFs predictions still contain some slight distortions around the spine metal implant in Fig. 4. These results visualize that the JBF, although enforcing proximity to the noisy input, is still dependent on a reasonable guidance image. This dependence is desired as learned information from the guidance image should be employed during filtering. Our experiments show that mainly artifacts where image content is entirely removed in large areas of the guidance image are difficult to restore through JBFs. Please note that, the shown artifacts introduced by the QAE network can be regarded as worst-case in a clinical pipeline and are still satisfactorily handled by the JBFs considering the original, unfiltered QAE predictions.

DL frameworks like *PyTorch*^34^ allow an automatic calculation of gradients in their operators. Therefore, one could think of implementing a JBF directly from *PyTorch* tensors instead of using analytical gradients to make its parameters trainable. Although this is possible, training such a filter would require expensive Python loops over the training batches and kernel windows which would accumulate huge computational graphs for the gradient calculation. In practice, training such a model with reasonable image and batch sizes, therefore, is infeasible in terms of computational time and GPU memory. The analytical filter derivative presented in this work greatly simplifies the required computations to enable data-driven optimization and limit the computational overhead through adding JBF layers as shown by comparing run times with convolutional layers. Eventually, we believe that our open-source filter layer can be useful in further hybrid applications as a known denoising operator that can be optimized in a data-driven manner.

## Conclusion

In this work, we presented a trainable JBF layer that can be incorporated into any deep model. We propose a hybrid denosing pipeline using these JBF layers and pre-trained deep denoising neural networks. The latter can produce faulty predictions when tested on data that is insufficiently represented in the training domain. In our experiments, we show that JBFs prevent DL-based models from severe prediction failures although the JBFs make use of distorted guidance images predicted from the neural networks. These results are explained by the clear algorithmic design of the JBF that limits the influence of the guidance image to the contribution of the intensity range filter kernel. We think that JBF layers can combine the flexibility of deep neural networks with the prediction reliability of conventional methods to leverage the power of deep models in clinical low-dose CT applications.

## Supplementary Information

Supplementary Information.

## Data Availability

The data sets analysed during the current study are publicly available in the *TCIA Low Dose CT Image and Projection Data* repository (Version 4)^35^, https://doi.org/10.7937/9NPB-2637. Coordinates and exemplary slices of all analyzed abdomen ROIs are included in the supplementary material.

## Code availability

The implementation of our open-source CUDA-accelerated trainable bilateral filter layer (*PyTorch*) together with example scripts and tests is publicly available at https://github.com/faebstn96/trainable-joint-bilateral-filter-source.
