# Supplementary material for: Trainable joint bilateral filters for enhanced prediction stability in low-dose CT
Source: Sci Rep. 2022 Oct 20;12:17540. doi: 10.1038/s41598-022-22530-4 (PMC9585057; doi:10.1038/s41598-022-22530-4)
Supplement: Supplementary file 1 — Supplementary Information. [file 41598_2022_22530_MOESM1_ESM.pdf]

Supplementary material: Trainable Joint Bilateral Filters for Enhanced Prediction Stability in Low-dose CT

Fabian Wagner[1,*], **Mareike Thies**[1], **Felix Denzinger**[1], **Mingxuan Gu**[1], **Mayank Patwari**[1], **Stefan Ploner**[1], **Noah Maul**[1], **Laura Pfaff**[1], **Yixing Huang**[1], **and Andreas Maier**[1]

[1]Pattern Recognition Lab, Friedrich-Alexander-Universität Erlangen-Nürnberg, 91058 Erlangen, Germany
[*]fabian.wagner@fau.de

Scan ID	x start	x end	y start	y end	z start	z end
L006	190	240	170	210	204	214
L033	150	380	95	130	0	11
L049	205	320	275	380	6	9
L058	450	500	230	300	205	209
L075	200	315	160	250	16	28
L077	215	300	455	480	7	9
L107	230	340	430	460	74	82
L114	460	500	190	250	10	15
L134	70	170	220	360	6	7
L148	35	240	135	260	0	38
L160	215	285	280	340	99	114
L170	175	330	205	340	37	115
L178	105	185	245	310	0	7
L187	210	300	375	420	33	45
L193	200	305	175	250	161	168
L203	200	415	240	325	11	55
L232	210	270	270	310	151	159

Table 1. Coordinates of the 17 investigated abdomen ROIs in scans of the *TCIA Low Dose CT Image and Projection* data set (Version 4)[1]. ROIs are defined by their start and end pixel index (starting from 0) in all three dimensions. Figure 1 illustrates exemplary CT slices that contain each ROI.

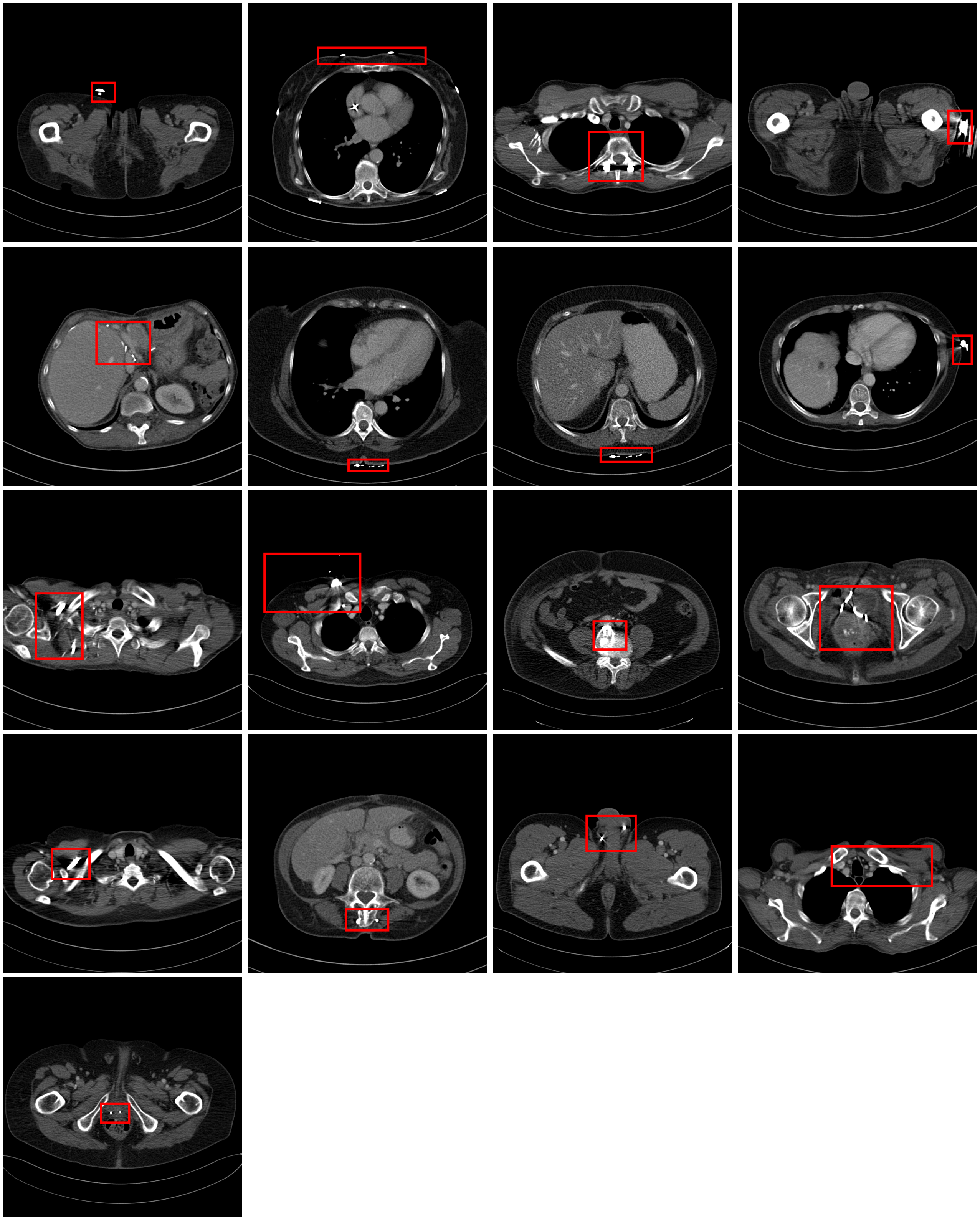

Figure 1. Exemplary low-dose input slices with all 17 selected 3D abdomen ROIs (highlighted in red) that contain metal parts. The patients from Table 1 are plotted from left to right and from top to bottom. Note that all ROIs are chosen around three-dimensional objects such that metal pieces are not always found in the center of some of the displayed ROIs. The reconstruction window is $[-150, 500]$ HU.

References

1. Moen, T. R. *et al.* Low-dose CT image and projection dataset. *Med. Phys.* **48**, 902–911, DOI: https://doi.org/10.1002/mp.14594 (2021).
